# Differentiation of West Nile and Usutu Virus Infections by Antibodies Directed to the Non-Structural Protein 1

**DOI:** 10.3390/v17101357

**Published:** 2025-10-10

**Authors:** Lena Roßbacher, Samuel Taschler, Elena Cecchettin, Amelie Popovitsch, Stephan W. Aberle, Judith H. Aberle, Iris Medits-Weiss, Karin Stiasny

**Affiliations:** Center for Virology, Medical University of Vienna, 1090 Vienna, Austria; lena.rossbacher@meduniwien.ac.at (L.R.); elena.cecchettin@meduniwien.ac.at (E.C.); amelie.popovitsch@meduniwien.ac.at (A.P.); stephan.aberle@meduniwien.ac.at (S.W.A.); judith.aberle@meduniwien.ac.at (J.H.A.)

**Keywords:** West Nile virus, Usutu virus, non-structural protein 1, orthoflavivirus cross-reactivity, orthoflavivirus serodiagnosis

## Abstract

The genus *Orthoflavivirus* (family *Flaviviridae*) comprises several important pathogens that are widespread across the globe, often co-circulating in many regions. In Austria, the closely related mosquito-borne West Nile (WN) and Usutu (USU) viruses have been detected since the early 2000s. Orthoflavivirus-neutralizing antibodies primarily target the major envelope protein E. However, due to their antigenic relationship, recurring contacts with different orthoflaviviruses can lead to the induction of broadly cross-reactive E-specific antibodies. These can pose a problem in the diagnosis and differentiation of orthoflavivirus infections. Therefore, we established immunological assays based on the non-structural protein 1 (NS1) to differentiate infections caused by WN and USU viruses. The NS1 protein is secreted during acute infection, and NS1-specific antibodies have been reported to be less cross-reactive than those against E. Using sera from individuals with a confirmed WN or USU virus infection, it was possible to distinguish between the two virus infections with high accuracy, specifically when IgM and IgG results were combined.

## 1. Introduction

West Nile (WN) and Usutu (USU) viruses are closely related mosquito-borne orthoflaviviruses that belong to the Japanese encephalitis serocomplex [1]. WN virus (WNV) and USU virus (USUV) originated in Africa [2,3], with WNV being now almost worldwide distributed. Since their discovery, both viruses have become endemic in many regions of Central Europe [4,5], with co-circulation in the northeastern part of Austria since the early 2000s [6]. WNV was found in wild animals in 2008 [7]; the first human case occurred the following year [8]. USUV was identified in dead blackbirds after a sudden mass mortality in 2001 [4]. In 2017, the virus was detected in several blood donors [9]. WNV and USUV are transmitted usually to humans via bites from infected mosquitoes, but it is important to note that WNV can also be acquired through blood transfusions [10]. As viremia in humans is usually rather short and low, they are generally considered to be dead-end hosts [11].

WN and USU viruses can cause a variety of different symptoms, with around 80% of WNV and most USUV infections being asymptomatic [12,13]. Symptomatic WN disease ranges from WN fever (WNF) to severe neurological manifestations (WN neuroinvasive disease, WNND) [14]. Symptomatic human USUV infections are rare and mainly affect immunocompromised people [15]. Diagnosis can be difficult because of the short viremia and unspecific symptoms [16]. Early after infection, PCR gives specific results, but later in the disease, only serological diagnosis is possible. The primary target for orthoflavivirus-induced antibodies is the major envelope protein E [17], but these antibodies can be cross-reactive, particularly for closely related orthoflaviviruses such as WNV and USUV [18].

Current serological tests mainly detect E-specific IgM and IgG antibodies, and orthoflavivirus cross-reactive antibodies reduce the specificity of these tests [18,19]. This problem is exacerbated by a number of factors, including habitat expansion and the introduction of new orthoflaviviruses into previously unaffected areas [20]. Increased viral co-circulation, the use of different orthoflavivirus vaccines and travel to orthoflavivirus-endemic countries contribute to the complexity of accurate diagnosis, as sequential exposure to different orthoflaviviruses promotes the production of cross-reactive E-specific antibodies [21,22].

The non-structural protein 1 (NS1) is a well-described alternative antigen for the serodiagnosis of orthoflavivirus infections [23]. It is found intracellularly, extracellularly, as a membrane-bound dimer, or is secreted as higher-order oligomers [24]. Experiments with recombinantly produced NS1 revealed the presence of dimers, tetramers and/or hexamers, suggested to be in a dynamic equilibrium, affected by a variety of conditions, such as the virus and/or strains used, the expression cell line, and the purification schemes [24]. NS1 is a multifunctional protein, with a key role in viral replication [25]. Furthermore, it is involved in immune evasion and has been recognized as a pathogenesis factor for dengue [26], the most prevalent orthoflavivirus disease. Both membrane-bound and secreted NS1 are immunogenic and induce antibody responses [27].

As NS1-specific antibodies have been reported to be less cross-reactive than E-specific antibodies [23], we investigated whether NS1-based IgM and IgG ELISAs using recombinant antigens allow a differentiation between WN and USU virus infections. Our study shows that NS1-based antibody detection has indeed the potential to improve the reliability of serological diagnosis of infections caused by these two closely related orthoflaviviruses.

## 2. Materials and Methods

### 2.1. Human Serum Samples

Serum samples of 18 WN and 17 USU PCR-confirmed cases were available that originally had been submitted for diagnostic purposes to the Center for Virology of the Medical University of Vienna (Table 1). In addition, samples from nine PCR-negative WN patients were analyzed (Table 2). Twenty-three of the WN and all USU cases have already been described in earlier epidemiological studies [6,8,28].

The 27 WN cases were diagnosed according to the ECDC’s official WN case definition as outlined in the Commission Implementing Decision (EU) 2018/945 (https://eur-lex.europa.eu/eli/dec_impl/2018/945/oj/eng; accessed on 19 August, 2025). A confirmed WNV case is defined by one of the following criteria: (i) detection of WNV-specific nucleic acid in blood, cerebrospinal fluid (CSF), or urine; (ii) detection of WNV-specific IgM in CSF; or (iii) high-titer WNV-specific IgM in serum combined with detection of WNV-specific IgG, confirmed by virus-specific neutralization testing.

Among the 18 PCR-confirmed WN cases, 13 were symptomatic infections and five were detected by regular screening of blood donations (Table 1). All nine WN cases identified through serology were symptomatic infections (Table 2). Of the 22 WN patients (Table 1 and Table 2), two had WNND and eight had WNF; the disease course of the other patients was not reported to the diagnostic laboratory. Three of the blood donors remained asymptomatic, while one experienced fatigue and one developed WNF.

The USU cases were infections detected in 16 blood donors who did not show symptoms and one patient with neuroinvasive disease (Table 1). In the case of the USU patient, three serum samples were available. Upon hospitalization, both serum and cerebrospinal fluid (CSF) were tested using USUV-specific PCR, and USUV RNA was detected in the CSF sample [28].

In addition, 20 tick-borne encephalitis (TBE) NS1 IgG-positive sera from a previous study were used [29].

### 2.2. PCR and Neutralization Tests (NTs)

PCR testing was performed as described previously [8,9]. Briefly, RNA extraction was done with the NucliSENS easyMAG extractor (bioMérieux, Marcy l’Etoile, France), followed by RT-PCR.

NTs were carried out, as described previously [6,8]. Duplicates of serial twofold dilutions of heat-inactivated serum samples were incubated with 50–100 Tissue Culture Infectious Doses 50% (TCID50) of WNV (strain NY99) or USUV (South African strain SAAR-1776) for 1 h at 37 °C. Vero cells were added and incubation was continued for 4–5 days. Viral replication was assessed by the occurrence of a cytopathic effect. Neutralization titers ≥20 were considered positive.

### 2.3. NS1 Protein Production and Purification

The recombinant WN and USU NS1 proteins, each containing a C-terminal Strep-tag, were based on the following gene sequences (synthesized by GeneArt, Regensburg, Germany; Thermo Fisher Scientific, Waltham, MA, USA): WN, GenBank accession no. KM659876; USU, GenBank accession no. HM569263. They were codon optimized for expression in the S2 Drosophila expression system, as previously described for TBE NS1 (GenBank accession no. TBE, U27495) [29]. Briefly, Schneider S2 Drosophila cells were stably transfected with the respective expression plasmid and a blasticidin selection vector. Protein expression was induced by the addition of CuSO_4_. The cell culture supernatant containing the recombinant NS1 proteins was harvested seven to eleven days after transfection. NS1 proteins were purified by Strep-Tactin-affinity chromatography (IBA Lifesciences GmbH, Göttingen, Germany), according to the manufacturer’s instructions. Protein concentrations were determined with the Pierce BCA Protein Assay (Thermo Fisher Scientific, Waltham, MA, USA) following the manufacturer’s protocol. Protein concentration and purity were verified by sodium dodecyl sulfate-polyacrylamide gel electrophoresis (SDS-PAGE) according to Laemmli [30].

### 2.4. Determination of the Oligomeric State of NS1

The oligomeric state of the WN and USU NS1 proteins was assessed using three different methods, with each method carried out in two independent experiments.

Five hundred ng of purified protein were analyzed by SDS-PAGE under native and reducing conditions using 4–20% Mini-PROTEAN^®^ TGX™ Precast Protein Gels (Bio-Rad Laboratories Inc., Hercules, CA, USA), according to the manufacturer’s instructions.

Five μg of purified protein were cross-linked with 10 mM dimethyl-suberimidate solution (DMS, Pierce, Thermo Fisher Sceintific, Waltham, MA, USA). After a 30 min incubation at room temperature (RT), the reaction was stopped with ethanolamine (Merck Chemicals and Life Science GesmbH, Vienna, Austriak) at a final concentration of 10 mM, and incubated at RT for 15 min. The protein was solubilized with 1.5% sodium deoxycholate (Sigma-Aldrich, St. Louis, MO, USA) and precipitated with 100% trichloroacetic acid (TCA). The cross-linked proteins were analyzed by SDS-PAGE according to Maizel [31], using a 5% phosphate gel under non-reducing conditions.

One hundred µg of purified protein were subjected to analytical size-exclusion chromatography (SEC) using a Superdex 200 Increase 10/300 GL column (Cytiva, Marlborough, MA, USA), according to the manufacturer’s instructions.

### 2.5. NS1 IgM ELISA

Two hundred ng/well NS1 in carbonate coating buffer pH 9.6 was applied overnight to untreated 96-well microtiter plates (Nunc, Thermo Fisher Scientific, Waltham, MA, USA). After the coating buffer was removed, the plates were incubated for 30 min at 37 °C with blocking buffer (PBS pH 7.4, 1% BSA, 0.05% Tween 20). The serum samples were pretreated with IgG/Rheumatoid factor (RF) absorbent (Eurosorb from Euroimmun Medizinische Labordiagnostika AG, Lübeck, Germany)), according to the manufacturer’s instructions. The pre-treated samples were then serially diluted in ELISA buffer (PBS pH 7.4, 2% sheep serum, 2% Tween 20), applied to the plates and incubated at 37 °C for 45 min. Bound IgM antibodies were detected with a goat anti-human IgM horseradish peroxidase (Thermo Fisher Scientific, Waltham, MA, USA). 5′-Tetramethylbenzidine (TMB) substrate solution (Thermo Fisher Scientific, Waltham, MA, USA) was used according to the manufacturer’s instructions. The reaction was stopped after 20 min by adding 1 M sulfuric acid. Absorbance was measured at 450 nm using a Biotek Synergy HTX ELISA reader (Agilent, Santa Clara, CA, USA).

### 2.6. NS1 IgG ELISA

One hundred ng/well NS1 in carbonate coating buffer pH 9.6 was applied overnight to untreated 96-well microtiter plates (Nunc, Thermo Fisher Scientific, Waltham, MA, USA). After the coating buffer was removed, the plates were incubated for 30 min at 37 °C with blocking buffer. Next, serial dilutions of human sera in ELISA buffer were added to the plates, and incubated at 37 °C for 45 min. Bound IgG antibodies were detected with a goat anti-human IgG horseradish peroxidase (Thermo Fisher Scientific, Waltham, MA, USA). The remaining steps were carried out as described for the IgM ELISA.

### 2.7. Data Analysis and Statistics

Titers were determined by curve fitting with a logistic regression with four parameters using GraphPad Prism 10 (GraphPad Software, Boston, MA, USA). The cut-off value was calculated from the mean absorbance at the initial dilution plus three standard deviations of eight orthoflavivirus-negative diagnostic serum samples from previous studies [32]. The titers shown are geometric mean titers of at least two independent experiments.

Antibody titers below the detection limit of the assay were assigned values equal to 50% of this limit for data analysis. ELISA titers <100 were therefore considered 50.

Statistical analyses were performed with GraphPad Prism 10 (GraphPad Software, Boston, MA, USA). Significances between two groups were determined with Wilcoxon matched pairs tests. Friedman tests followed by Dunn’s multiple comparison tests were used for the comparison of more than two groups. Correlation coefficients were determined with the Pearson correlation test using logarithmic-transformed titers. *p*-values ≤ 0.05 were considered significant.

## 3. Results

### 3.1. Characterization of Recombinant WN and USU NS1 Proteins

**Figure 1:**
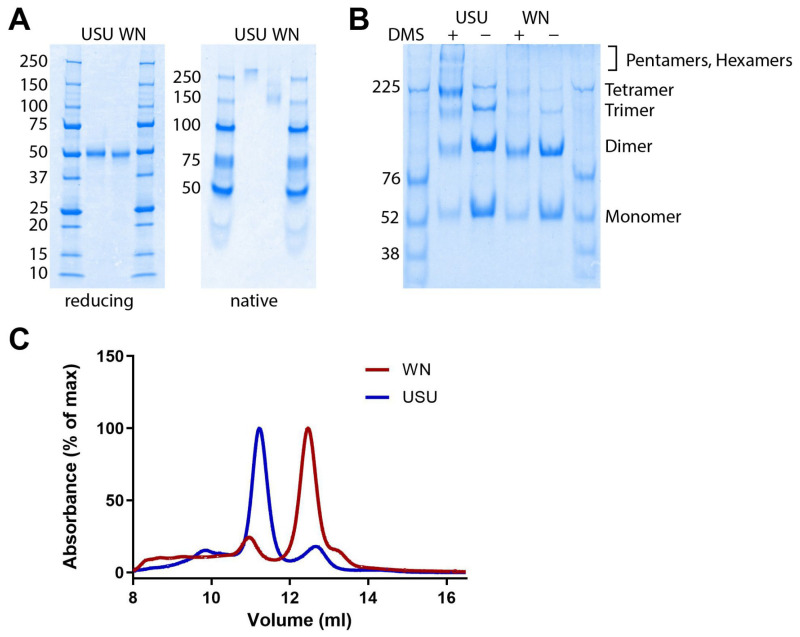
Characterization of recombinant USU and WN NS1 proteins produced in Drosophila S2 cells. (**A**) SDS-PAGE under reducing (**left**) and native (**right**) conditions. (**B**) Chemical cross-linking of NS1 proteins. (+) Cross-linked proteins; (−) non-treated controls. The gels were stained with Coomassie blue. The molecular weight (kDa) of the marker proteins is indicated on the left side of the gels. (**C**) Size-exclusion chromatography (SEC) of WN (red) and USU (blue) NS1 proteins. The elution volume of USU NS1 around 11.2 mL and the elution volume of WN NS1 around 12.5 mL correspond to a molecular weight of about 240–300 kDa and 120–130 kDa, respectively. *X*-axis: elution volume in ml. *Y*-axis: measured absorbance values shown as percentage of the maximum absorbance. WN, West Nile; USU, Usutu.

The recombinant WN and USU NS1 proteins were produced with the Drosophila expression system and purified by affinity chromatography, as described in Materials and Methods. The proteins were >90% pure and had a molecular weight of ~50 (50–55) kDa, as assessed by SDS-PAGE under denaturing and reducing conditions (Figure 1A, left panel). The slight difference in migration behavior could be due to variations in the glycosylation of the two proteins. SDS-PAGE under native conditions showed that the NS1 proteins formed higher-order oligomers, as reported for several orthoflaviviruses (Figure 1A, right panel) [24].

The oligomeric structure was confirmed by cross-linking (Figure 1B) and SEC (Figure 1C). The USU NS1 protein formed more stable, higher-order oligomers (tetramers and/or hexamers) than the WN NS1 protein, which was mostly present as dimers that may have been higher-order oligomers that had dissociated under different experimental conditions (Figure 1A, right panel; Figure 1C).

### 3.2. Detection of NS1 IgM and IgG Antibodies in Serum Samples of WN and USU Cases

The main objective of this study was to analyze WN-USU cross-reactivity of NS1-specific antibodies in IgM and IgG ELISAs, and to assess their potential as additional diagnostic tools for cases where PCR testing has returned a negative result. For this purpose, we used samples from confirmed WN and USU cases (see Section 2) with a positive titer in the respective NT for our assays (Tables S1 and S2).

First, we quantified WN and USU NS1-specific IgM and IgG antibodies in serum samples from PCR-confirmed WNV- and USUV-infected individuals (Table 1, Tables S1 and S2). The WN cohort consisted of 13 symptomatic patients and 5 asymptomatic blood donors; the USU cohort included the 16 asymptomatic blood donors (Table 1). The WN samples were obtained between 0 and 44 days after the positive PCR result (days post PCR, dpPCR) with a median of 11 dpPCR. The USU samples were collected between 11 and 48 dpPCR, with a median of 24 dpPCR.

**Figure 2:**
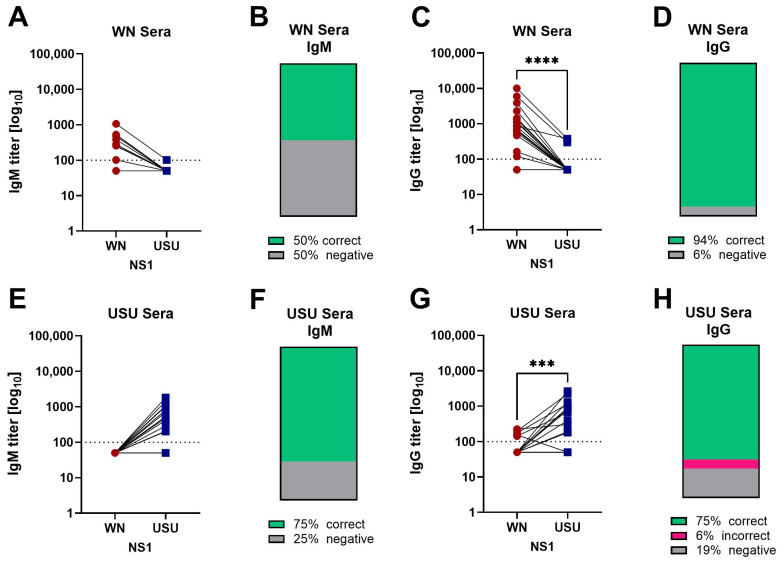
NS1-specific antibody titers in serum after WNV and USUV infection. IgM (**A**) and IgG (**C**) ELISA titers from PCR-confirmed WN (*n* = 18) cases. IgM (**E**) and IgG (**G**) ELISA titers from PCR-confirmed USU (*n* = 16) cases. Titers were determined with WN (red circles) and USU (blue squares) NS1 antigens. (**B**,**D**,**F**,**H**) Categorization of sera according to the ELISA titers obtained with both antigens: percentage of correctly classified sera in green, incorrectly classified sera in magenta, negative sera in grey. Significances were determined with the Wilcoxon matched pairs test, and are indicated by asterisks (**** *p* < 0.0001, *** *p* = 0.001 to 0.0001). Dotted line: cut-off of the assays. WN, West Nile; USU, Usutu.

Nine sera from the WN cohort (50%) tested positive in the WN NS1 IgM ELISA, with significantly higher titers against WN NS1 than against USU NS1 (Figure 2A,B). Seventeen of the 18 sera were negative in the USU NS1 IgM ELISA (Figure 2A,B). Seventeen sera (94%) reacted with WN NS1 in the IgG ELISA (Figure 2C,D). Of these WN IgG-positive samples, 14 exhibited no reactivity in the USU NS1 IgG ELISA. Overall, the WN NS1 IgG titers were significantly higher than the USU NS1 IgG titers. One WN serum showed no reactivity in the WN and USU IgG assays.

Twelve sera from the USU cohort (75%) were positive in the USU NS1 IgM ELISA (Figure 2E,F), and all USU sera were WN IgM negative (Figure 2E,F). USU NS1 IgG antibodies were detected in 12 sera (75%), and the USU NS1 IgG titers were significantly higher than the WN NS1 IgG titers (Figure 2G,H). Eight of the twelve USU NS1 IgG-reactive sera were negative in the WN NS1 IgG ELISA (Figure 2G,H). One USU serum showed low-level IgG reactivity with WN NS1, but not with USU NS1 (USU case 5 in Table S2), and had no IgM reactivity with any NS1 antigen. In this case, a follow-up sample would have been helpful to monitor possible changes in titers over time. Another USU serum had similar low reactivities in the WN and USU NS1 IgG ELISA, but only USU NS1-specific antibodies were detected in the IgM ELISA (USU case 1 in Table S2).

Overall, these data suggest that it is possible to differentiate between WN and USU virus infections using a combination of IgM and IgG NS1 ELISAs. However, caution should be exercised with low-titer results, as these appear to be less accurate.

**Figure 3:**
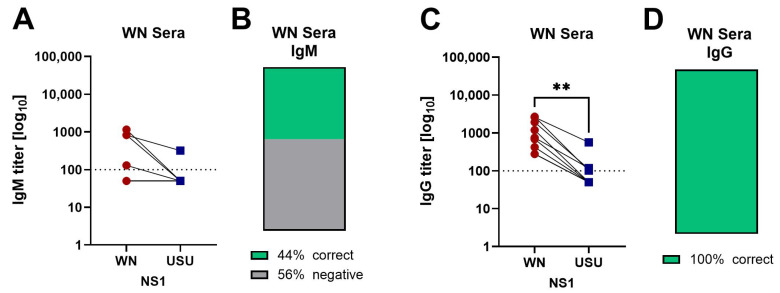
NS1-specific antibody titers in serum after WNV infection. (**A**) IgM and (**C**) IgG ELISA titers from serologically confirmed WN cases (*n* = 9). Titers were determined with WN (red circles) and USU (blue squares) NS1 antigens. (**B**,**D**) Categorization of sera according to the ELISA titers obtained with both antigens: percentage of correctly classified sera in green, negative sera in grey. Significances were determined with the Wilcoxon matched pairs test, and are indicated by asterisks (** *p* = 0.01 to 0.001). Dotted line: cut-off of the assays. WN, West Nile; USU, Usutu.

Next, sera from nine symptomatic WN patients with negative PCR results and serological diagnoses were analyzed (Table 2). The samples were obtained at a median of eight days after hospitalization (range 0–27 days). Four samples (44%) reacted in the WN NS1 IgM ELISA, and the titers were higher than in the USU NS1 IgM ELISA, with three of the four sera testing negative against USU NS1 (Figure 3A,B). All WN sera (100%) had detectable levels of WN NS1-specific IgG antibodies. Four sera cross-reacted with USU NS1, but the WN NS1 IgG titers were significantly higher than the USU NS1 IgG titers (Figure 3C,D). Altogether, our results are in agreement with the previous serological WN diagnosis.

**Figure 4:**
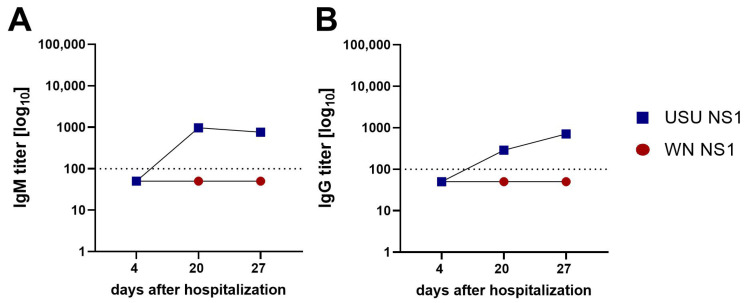
NS1-specific antibody titers in serum from one serologically confirmed USUV-infected patient. (**A**) IgM and (**B**) IgG ELISA titers from sera taken at different days post disease onset. Titers were determined with WN (red circles) and USU (blue squares) NS1 antigens. Dotted line: cut-off of the assays. WN, West Nile; USU, Usutu.

Symptomatic USU disease is rare, and samples were available from only one USU patient with neuroinvasive disease, which has been described in detail in a previous report [28]. We tested three sequential samples in the WN and USU IgM and IgG NS1 ELISAs. On day four after hospitalization, all IgM and IgG ELISAs were still negative (Figure 4A,B). On day 20, USU NS1-specific IgM and IgG antibodies were detected. At the last sampling time point (27 days after hospitalization) the USU IgM titer was slightly lower, while the USU IgG titer was still increasing. Furthermore, none of the samples reacted with WN NS1 at any time, confirming the USU diagnosis.

### 3.3. Comparison of WN and USU NS1 IgG and NT Titers

To analyze the relationship between WN and USU NS1 ELISA titers and the NT titers used for sample selection (Tables S1 and S2), we evaluated all WN (*n* = 27) and USU (*n* = 17) cases for positivity in both assay formats. As can be seen in Table 3, strong cross-neutralization was detected, with 85% of the WN samples testing positive in the USU NT, and 65% of the USU samples in the WN NT. Cross-reactivity in the NS1 ELISAs was lower, 0–7% in IgM assays and 26–35% in IgG assays.

Most samples had a higher titer in the NT with the infecting virus than with the heterologous virus. However, three PCR-confirmed cases did not follow this pattern (Table 4), showing equal titers in both NTs or even a higher titer in the heterologous NT. In contrast, the WN and USU NS1 IgM and IgG reactivities allowed a correct identification (Table 4).

Overall, the NS1 ELISAs showed less WN-USU cross-reactivity than the NTs, with the exception of one USU case (see Section 3.2) that had only a low titer in the WN NS1 IgG ELISA and was negative in the USU NS1 ELISAs (USU case 4 in Table S2, USU NT titer 40, WN NT titer <20).

### 3.4. Detection of Broadly Cross-Reactive Antibodies in WN and USU ELISAs

As shown in Figure 2 and Figure 3 as well as Table 3, cross-reactive binding of WN and USU sera to the respective heterologous antigens was observed. This was mainly in the IgG tests and to a lesser extent in the IgM tests. In several European countries, including Austria, another orthoflavivirus, tick-borne encephalitis virus (TBEV), is endemic, and co-circulates with WNV and USUV [33]. Since TBEV is more distantly related to WNV and USUV, with an amino acid identity of NS1 proteins of only ~40% (Figure S1), we investigated whether broad cross-reactivity might interfere with our WN and USU assays. We therefore analyzed 20 samples from TBE patients, already shown to be TBE NS1 IgG-positive in a previous study [29].

**Figure 5:**
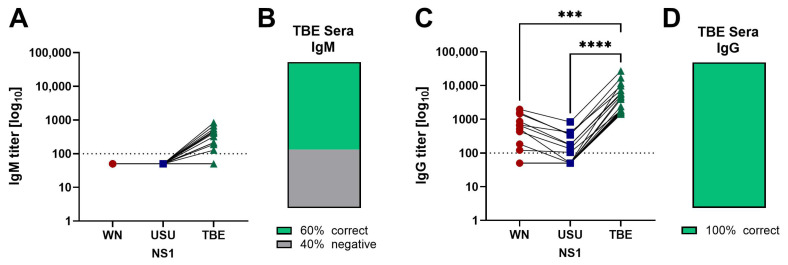
NS1-specific antibody titers in serum after TBEV infection. (**A**) IgM and (**C**) IgG ELISA titers of TBEV-infected patients (*n* = 20). The titers were determined with WN (red circles), USU (blue squares) and TBE (green triangles) NS1 antigens. (**B**,**D**) Categorization of sera according to the ELISA titers obtained with WN, USU and TBE NS1: percentage of correctly classified sera in green and negative sera in grey. Significances were determined with the Friedman test followed by Dunn’s multiple comparison test, and are indicated by asterisks (**** *p* < 0.0001, *** *p* = 0.001 to 0.0001). Dotted line: cut-off of the assays. WN, West Nile; USU, Usutu. TBE, tick-borne encephalitis.

Twelve TBE sera (60%) reacted in the IgM ELISA with the homologous TBE NS1 (Figure 5A,B), but cross-reactive binding to USU and/or WN NS1 proteins was not detected. As expected, TBE sera reacted with the homologous NS1 protein in the IgG ELISA. Cross-reactivity was observed with both heterologous NS1 proteins, but the WN and USU IgG titers were significantly lower than the TBE IgG titers (Figure 5C,D). Ten samples yielded a negative result (50%) in the WN IgG ELISA, 12 sera (60%) were negative in the USU IgG ELISA.

Percent cross-reactivity in the WN and USU NS1 IgG ELISAs was slightly higher with the TBE sera than with the WN and USU sera (Table 3). As the TBE cohort contained less low-titer sera than the WN and USU cohorts (compare Figure 2, Figure 3, Figure 4 and Figure 5), we performed correlation analyses to assess whether the magnitude of the homologous NS1 response influenced cross-reactivity results.

**Figure 6:**
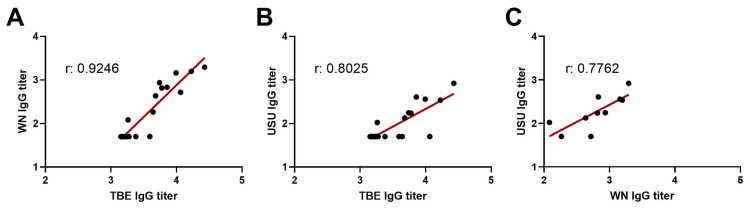
Correlations of different NS1-specific IgG titers of TBE sera. (**A**) TBE and WN IgG ELISA titers of TBE sera, (**B**) TBE and USU IgG ELISA titers of TBE sera, (**C**) WN and USU IgG ELISA titers of TBE sera. Double-negative sera are not included. The Pearson correlation coefficient (r) is indicated in each graph. The linear regression line is shown in red. WN, West Nile; USU, Usutu. TBE, tick-borne encephalitis.

There were significant positive correlations (*p* < 0.0001) between TBE IgG titers and WN or USU IgG titers of the TBE sera (Figure 6A,B). A significant positive correlation (*p* = 0.008) was also found when we compared the WN and USU IgG titers of the TBE sera (Figure 6C). These results indicate that broadly cross-reactive antibodies, which represent only a small fraction of the total antibody response, are mainly detectable in sera with high titers against the homologous TBE NS1 protein.

## 4. Discussion

Correctly identifying orthoflavivirus-specific antibodies is essential for serodiagnosis and disease surveillance, which, however, can be challenging due to the broad cross-reactivity of E protein-specific antibodies. Although the NT is highly specific and is considered the gold standard, cross-neutralization between members of the same serocomplex, like WNV and USUV [1], can make it difficult to differentiate between such closely related viruses. A rather recent approach for improving serodiagnosis is the detection of antibodies targeting NS1, which are reported to be less cross-reactive than E protein-specific antibodies [34].

We successfully established quantitative IgM and IgG ELISAs that allowed the differentiation of WNV and USUV infections with high accuracy. Altogether, the IgM ELISAs had lower sensitivity but higher specificity than the IgG assays, which has also been reported for dengue, Zika and TBE NS1-based tests for human sera [35,36,37]. The low sensitivity of IgM assays might be due to NS1-specific IgM antibodies representing only a small fraction of the total IgM response. As shown with a dengue IgM capture assay, the depletion of antibodies directed to the surface proteins increased the detection of NS1-specific IgM antibodies [35]. Further studies could investigate the effectiveness of this format. Another possible factor may be the formation of immune complexes of IgM antibodies with NS1, which is secreted into the blood during acute infection [38]. Such complexes may render NS1-bound IgM antibodies unable to be detected by ELISA.

The WN and USU NS1 IgG ELISAs had higher sensitivity than the IgM ELISAs, but we detected more antibodies that cross-reacted with WNV or USUV (Figure 2, Table 3) as well as with TBEV (Figure 5), a more distantly related orthoflavivirus (Figure S1). In particular, TBE sera with high TBE NS1 IgG titers yielded positive results in the WN and USU NS1 IgG assays (Figure 6). These titers, however, were significantly lower than the homologous titers (Figure 5 and Figure 6). Similar to our results, cross-reactivity with WN NS1 was found in a study using canine sera, in which high-titer TBE sera exhibited some WN NS1 cross-reactivity [39]. NS1 cross-reactive binding between both closely and distantly related orthoflaviviruses was also reported in a study with mouse sera [40]. USU sera reacted with WN NS1, and to a lesser extent, with dengue NS1 in the IgG ELISA. In regions, where several orthoflaviviruses co-circulate, NS1 IgG ELISAs should therefore preferably be performed with all corresponding NS1 proteins to allow a clear differentiation. In addition, a combination with the more specific NS1 IgM ELISAs could be considered.

Here, we evaluated NT-positive serum samples (Tables S1 and S2), collected at different time points after infection, reflecting typical conditions in a diagnostic setting. Most samples yielded higher NT titers for the infecting virus than for the heterologous virus, with three exceptions, but they were identified correctly with the NS1 assays (Table 4). Based on these findings, we would recommend NS1-based antibody assays as additional tests to complement NTs and/or E-based ELISAs, which is important for samples collected too late for PCR. Furthermore, NTs are time-consuming, requiring specialized laboratories (in the case of WNV even BSL3 facilities) and causing high costs. As NS1 ELISAs offer a faster and more cost-effective alternative, a two-step strategy may be considered for PCR-negative samples, starting with the cheaper NS1-based ELISAs, followed by NTs for samples that remain NS1 negative, near the cut-off, or inconclusive.

The first commercially available, widely used NS1-specific antibody kits for detecting IgM and IgG antibodies were for Zika. These kits produced promising results when samples from primary infections were analyzed [36]. However, in Zika-endemic regions with multiple orthoflaviviruses co-circulating, especially dengue viruses, cross-reactivity can limit the reliability of such assays [41]. For WNV, an NS1 IgG ELISA is commercially available. When compared to a WN NT, the commercial assay identified only 14 of 37 NT-positive samples [42]. Interestingly, the same group found that 35 of the 37 NT-positive samples were positive in their in-house WN NS1 IgG ELISA [42], in line with our results (Table 3). The low sensitivity of the commercial test remained unclear [42], but differences in assay set-ups and/or cut-offs might explain the discrepant results. In the case of dengue, a commercial NS1 IgG ELISA with proteins from all four serotypes has been evaluated for determining the pre-immune status in people considering dengue vaccination [43]. While the assay had a high sensitivity, some cross-reactivity was observed with samples after Zika virus-infection [43]. Notably, the authors did not recommend the dengue NS1 kit as a stand-alone test for their purpose. Instead, they also suggest a two-test approach, combining the NS1 ELISA with a dengue IgG rapid test using the envelope proteins of the four dengue virus serotypes [43].

Our serum samples likely originated from primary orthoflavivirus infections and, in most instances, we could clearly distinguish WNV and USUV infections using quantitative titer comparisons with NS1-based ELISAs. Vaccination history for orthoflaviviruses was not reported to the diagnostic laboratory for the cases tested, except for the USU patient, which had never been vaccinated against TBE, Japanese encephalitis or yellow fever [28]. Due to the high TBE vaccination coverage in Austria, many individuals were likely vaccinated with a formalin-inactivated whole-virus vaccine. Trace amounts of NS1 were found in the two European TBE vaccines, and low levels of TBE NS1-specific IgG antibodies were detected in a few individuals who had received two or more vaccine doses, but NS1 cross-reactivity has not been evaluated [44]. In contrast, two studies did not detect TBE-NS1-specific IgG antibodies in TBE vaccinees using an ELISA [29,45]. Furthermore, NS1-specific priming and anamnestic NS1 antibody response were not observed in TBE vaccination breakthrough infections [29]. A strong influence of TBE vaccination on our comparative WN and USU NS1 IgG results is therefore not expected. However, broad NS1 cross-reactivity may pose problems when analyzing samples from sequential orthoflavivirus infections. Substantial levels of broadly cross-reactive NS1-specific antibodies were found in cases of sequential dengue-Zika virus infections or secondary dengue virus infections [41,46]. Further studies are needed to improve our understanding of NS1 antibody cross-reactivity and its impact on assay specificity.

An interesting finding from the IgG ELISAs using human TBE post-infection sera was that all samples exhibited slightly stronger cross-reactive binding to WN NS1 than to USU NS1. Due to the equally distant relationship of WN and USU NS1 to TBE NS1 (~60% amino acid differences, Figure S1), this discrepancy would not have been expected. Our recombinant USU and WN NS1 proteins, however, differed in their glycosylation and oligomeric states (Figure 1), which might be responsible for our results. The USU NS1 protein apparently forms more stable higher-order oligomers than WN NS1, which may result in less accessible cross-reactive binding sites in USU NS1. Recently published studies also suggest that the oligomeric structure of different recombinant orthoflavivirus NS1 proteins varies. For example, dengue type 2 [47] and Zika NS1 [48] proteins were found in two studies to be predominantly tetramers. There are also differences between recombinant and infection-derived dengue type 2 NS1 proteins. Contrary to expectations, the latter were mainly present as dimers, in association with HDL, and only small amounts were found to be hexameric [49]. A possible influence of the oligomeric structure of NS1 on polyclonal antibody formation must also be considered [49]. Chew et al. pointed out that the significance of the different oligomeric structures of NS1 has not yet been sufficiently investigated [24]. Therefore, research is required to determine the extent to which the oligomeric states of the recombinant and natural NS1 proteins match.

Another factor that can influence NS1-specific antibody responses is the amount of NS1 produced during an orthoflavivirus infection and its kinetics. The concentration of NS1 in serum samples from dengue or yellow fever virus-infected patients revealed a high variability during the acute phase, but also a strong individual variation within patient cohorts [38,50,51]. The best studied orthoflavivirus in that respect is dengue virus, with NS1 levels ranging from undetectable up to ~8 µg/mL [38] and a maximum of 50 µg/mL reported for one case [50]. Primary infections are associated with higher NS1 serum levels than secondary infections, and an inverse correlation has been observed with NS1-specific IgG, suggesting the formation of NS1-IgG immune complexes or IgG-mediated NS1 clearance from circulation [38]. Comparable data are not available for WN and USU virus infections.

The limitations of our work were the small number of cases and the heterogeneity of the sampling time points. Studies using longitudinal acute and convalescent samples are therefore necessary for determining the most suitable testing window for NS1-specific antibodies. In addition, a precise analysis of NS1-antibody cross-reactivity would have required samples from sequential heterologous orthoflavivirus infections.

## 5. Conclusions

We demonstrated that differentiation between recent WNV and USUV infections is possible using quantitative WN and USU NS1-based IgM and IgG ELISAs, which should be performed side-by-side to minimize cross-reactivity effects. These tests represent valuable complementary tools for the serodiagnosis of PCR-negative cases, especially in regions where WN and USU viruses co-circulate, thus facilitating the surveillance of both virus infections. Furthermore, parallel testing could also provide insights into the epidemiology and clinical relevance of USUV.

## Figures and Tables

**Table 1:** Characteristics of PCR-confirmed cases.

Cases	*n* (Cases)	Patient/Blood Donor	Age (Median/Years)	Age (Range/Years)	Sex (f/m)
WN	18	13/5	57	36–77	5/13
USU	17	1/16	57	23–81	2/15

**Table 2:** Characteristics of serologically confirmed cases.

Cases	*n* (Cases)	Patient	Age (Median/Years)	Age (Range/Years)	Sex (f/m)
WN	9	9	67	35–87	5/4

**Table 3:** WN and USU NT and NS1 ELISA results of cases analyzed in this study.

Cases	*n*	WN NT	USU NT	WN NS1 IgM	USU NS1 IgM	WN NS1 IgG	USU NS1 IgG
		Number of Positive Cases/Total Number of Cases (% Positive Cases)					
WN	27	27/27 (100%)	22/26 (85%) *	13/27 (48%)	2/27 (7%)	26/27 (96%)	7/27 (26%)
USU	17	11/17 (65%)	17/17 (100%)	0/17 (0%)	13/17 (76%)	6/17 (35%)	13/17 (76%)

* One serum was not tested in the WN NT.

**Table 4:** WN and USU NT and ELISA titers from cases without conclusive NT results.

Cases *	Days After Positive PCR	WN NT	USU NT	WN NS1 IgM	USU NS1 IgM	WN NS1 IgG	USU NS1 IgG
		Titers					
WN 18	80	80	120	1062	101	1434	neg
USU 7	120	120	120	neg	197	1080	2247
USU 15	80	80	80	neg	neg	201	862

* See Tables S1 and S2.

## Data Availability

The original contributions presented in this study are included in the article/Supplementary Materials. Further inquiries can be directed to the corresponding authors.

## Supplementary Materials

The following supporting information can be downloaded at: https://www.mdpi.com/article/10.3390/v17101357/s1; Figure S1: Distance relationships between orthoflavivirus NS1 proteins based on amino acid sequence differences; Table S1: NT Titers of WN samples used in this study; Table S2: NT titers of USU samples used in this study.

## Abbreviations

The following abbreviations are used in this manuscript:

ELISA	Enzyme-linked immunosorbent assay
NS1	Non-structural protein 1
SEC	Size-exclusion chromatgography
TBE(V)	Tick-borne encephalitis (virus)
USU(V)	Usutu (virus)
WN(V)	West Nile (virus)
