# Supplementary material for: Differentiation of West Nile and Usutu Virus Infections by Antibodies Directed to the Non-Structural Protein 1
Source: Viruses. 2025 Oct 10;17(10):1357. doi: 10.3390/v17101357 (PMC12567941; doi:10.3390/v17101357)
Supplement: Supplementary file 1 [file viruses-17-01357-s001.zip › viruses-3789993-supplementary.pdf]

Supporting Figure

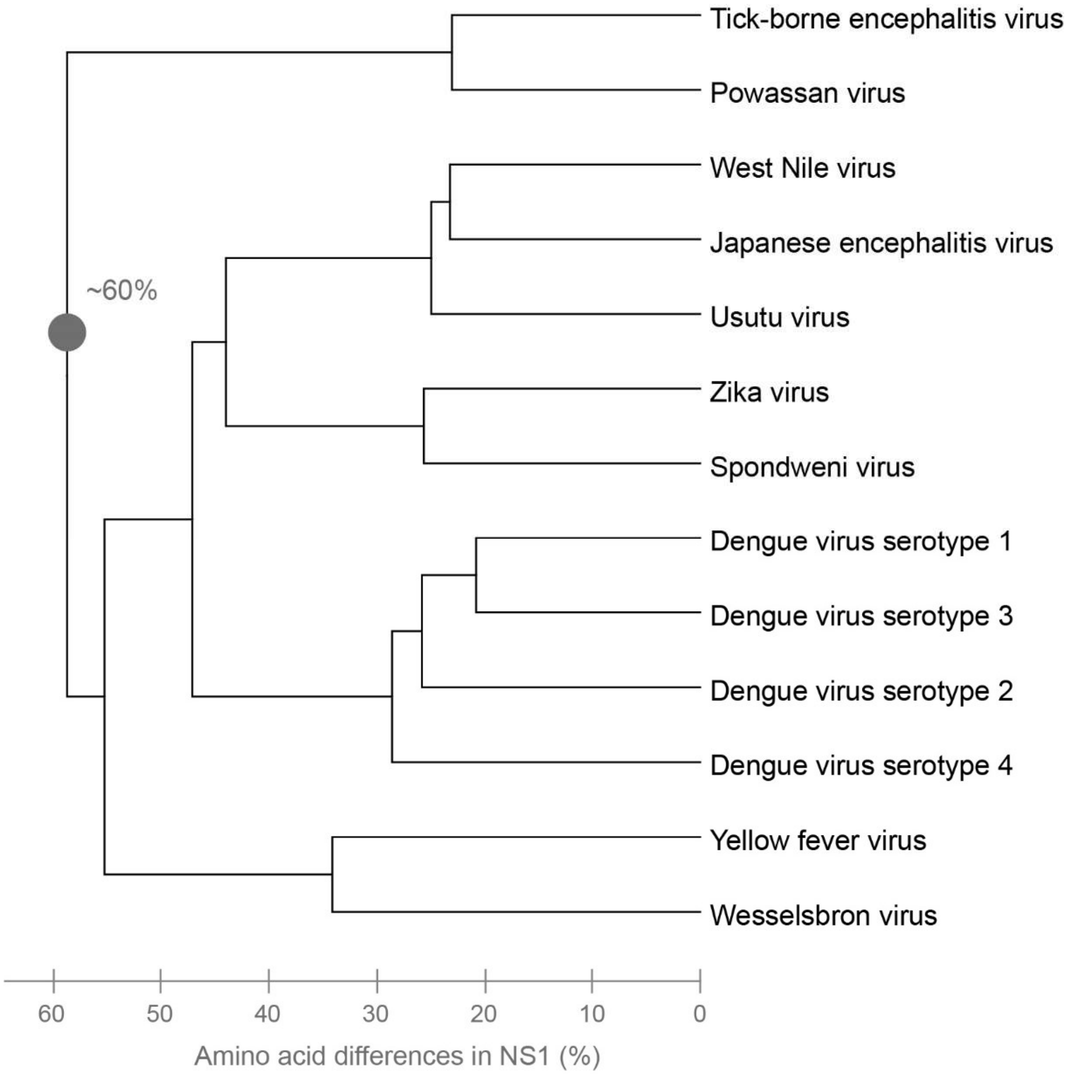

Figure S1. Distance relationships between orthoflavivirus NS1 proteins based on amino acid sequence differences. GenBank accession numbers of NS1 proteins are as follows: Tick-borne encephalitis virus (strain: Neudoerfl) U27495, Powassan virus (strain: LB) L06436, West Nile virus (strain: BD-AUT) KM659876, Japanese encephalitis virus (strain: SA14) D90194, Usutu virus (strain: Bologna-09) HM569263, Zika virus (strain: HPF-13) KJ776791, Spondweni virus (strain: SM_6_V_1) DQ859064, dengue 1 virus (strain: FGA/89) AF226687, dengue 3 virus (strain: CH5348) DQ863638, dengue 2 virus (strain: New_Guinea_C) M29095, dengue 4 virus (strain: DENV_4/SG/06K2270DK1/2005) GQ398256, yellow fever virus (strain: Asibi) AY640589, Wesselbron virus (strain: AV259) JN226796.

Supporting Tables

Table S1. NT titers of WN samples used in this study

	**WN NT**	**USU NT**	**days after positive PCR**	**days after hospitalization**
Blood donors				
**1**	1920	160	21	
**2**	240	80	30	
**3**	960	120	17	
**4**	160	60	31	
**5**	240	160	21	
PCR-confirmed WN patients				
**6**	1280	320	0	
**7**	960	160	11	
**8**	30	20	49	
**9**	320	80	5	
**10**	640	120	11	
**11**	240	160	0	
**12**	80	40	44	
**13**	320	40	8	
**14**	640	60	15	
**15**	240	<20	27	
**16**	160	80	12	
**17**	480	160	9	
**18**	80	120	8	
Serologically confirmed WN patients				
**19**	160	nd*		2
**20**	640	40		23
**21**	160	<20		8
**22**	320	120		8
**23**	80	20		0
**24**	480	60		3
**25**	320	<20		27
**26**	60	40		8
**27**	160	<20		0

* nd, not determined

Table S2. NT titers of USU samples used in this study

	WN NT	USU NT	days after positive PCR	days after hospitalization
Blood donors				
**1**	40	120	21	
**2**	<20	80	41	
**3**	40	80	17	
**4**	40	120	11	
**5**	80	240	19	
**6**	<20	40	26	
**7**	120	120	19	
**8**	<20	60	20	
**9**	80	120	23	
**10**	20	80	25	
**11**	30	120	20	
**12**	<20	160	42	
**13**	<20	40	39	
**14**	20	120	27	
**15**	80	80	48	
**16**	40	240	29	
Patient				
**17-1**	<20	20		4
**17-2**	<20	40		20
**17-3**	<20	40		27
